# The C-terminal sequence of the large hepatitis delta antigen is variable but retains the ability to bind clathrin

**DOI:** 10.1186/1743-422X-6-31

**Published:** 2009-03-16

**Authors:** Yu-Cheng Wang, Chi-Ruei Huang, Mei Chao, Szecheng J Lo

**Affiliations:** 1 Department of Microbiology, Graduate Institute of Biomedical Science, Chang Gung University, Kwei-Shan, Tao-Yuan 333, Taiwan; 2 Institute of Microbiology and Immunology, National Yang-Ming University, Taipei 112, Taiwan

## Abstract

**Background:**

Hepatitis delta virus (HDV) is a defected RNA virus and requires its encoded large antigen (LDAg) to interact with helper viral proteins (HBsAgs) during assembly. Recently, a study demonstrated a direct binding of the LDAg C-terminus from genotype I HDV to the clathrin heavy chain (CHC), which suggests that this interaction might facilitate HDV assembly. If LDAg binding to clathrin is essential to HDV life cycle, a clathrin box sequence at the C-terminus of LDAg should be conserved across all HDV. However, the C-terminal sequence of LDAg is variable among 43 HDV isolates.

**Results:**

Based on the presence and location of clathrin box at the C-terminus of LDAg from 43 isolates of HDV, we classified them into three groups. Group 1 (13 isolates) and 2 (26 isolates) contain a clathrin box located at amino acids 199–203 and 206–210, respectively, as found in genotype I and genotype II. Group 3 (4 isolates) contains no clathrin box as found in genotype III. CHC binding by three different LDAg (genotype I to III) was then tested by *in vivo *and *in vitro *experiments. Transfection of plasmids which encode fusion proteins of EGFP and full-length of LDAg from three genotypes into HuH-7 cells, a human heptoma cell line, was performed. GFP-pull down assays showed that a full-length of CHC was co-precipitated by EGFP-LDI, -LDII and -LDIII but not by EGFP. Further *in vitro *studies showed a full-length or fragment (amino acids 1 to 107) of CHC can be pull-down by 13-amino-acid peptides of LDAg from three genotypes of HDV.

**Conclusion:**

Both *in vivo *and *in vitro *studies showed that CHC can bind to various sequences of LDAg from the three major genotypes of HDV. We therefore suggest that the clathrin-LDAg interaction is essential to the HDV life-cycle and that sequences binding to clathrin are evolutionarily selected, but nonetheless show the diversity across different HDV genotypes.

## Background

Hepatitis delta virus (HDV) is a small defective RNA virus with a negative-stranded genome. It requires for a helper virus, hepatitis B virus (HBV), to supply envelope proteins (HBsAgs) to complete virion assembly and secretion [1-3]. The HDV genome is about 1,700 nucleotides long and is circular in form; it appears to form an unbranched rod-like structure due to a high degree of intra-molecular complementary base-pairing [4,5]. The genome sequence of HDV is divided into a viroid-like sequence and a protein-coding sequence [1,6]. It has been hypothesized that HDV resulted from RNA recombination between a viroid sequence and a cellular mRNA coding a DIPA (delta interacting protein) protein [6,7]. Analysis of HDV sequences from across world has revealed that those from Africa have the highest diversity, which suggests that the first HDV might have arisen in Africa [8,9]. After the additional isolation of new HDV sequences from Africa, the classification of HDV has been changed from one including genotypes I to III into one involving clades 1 to 8 [8].

**Figure 1:**
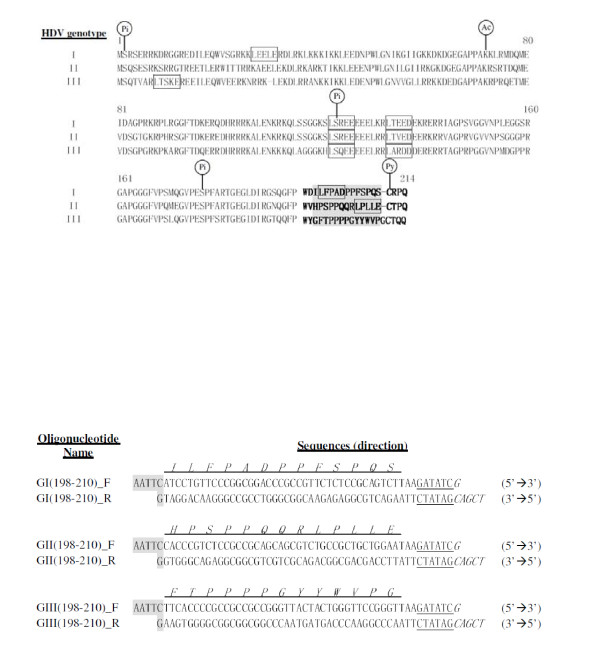
**The features of LDAg amino acid sequences of HDV and the synthetic oligonucleotides encoding the 13-amino-acid peptide of LDAg**. (A) The amino acid (in one-letter symbols) alignment of full-length of LDAg from the three genotypes. Genotype I is from American strain (accession number M28267), genotype II from Taiwan-3 strain (accession number U19598), and genotype III from Peru strain (accession number L22063). The putative clathrin binding domains are shown by rectangle boxes and the last 19 or 20 amino acids of LDAg are highlighted by bold face letters. The consensus amino acids for post-translational modification are indicated as follows: Ac (acetylation) at amino acid 72, Pi (phosphorylation) at positions of 2, 123, and 177 and Py (isoprenylation) at the position 211. The amino acid positions are indicated by numbers. (B) Two complementary oligonucleotides were designed for expression of the corresponding short amino acid sequences (as indicated in one-letter symbols above the oligonucletides) of LDAg. The 5'- and 3'-end of the oligonucleotides were designed to include the restriction sites *Eco*RI and *Sal*I, respectively. The restriction site immediately next to *Sal*I is *Eco*RV, which was designed specifically to allow quick clone selection.

In the past three decades, intensive molecular biology studies have largely revealed the functions and roles of HDV encoded proteins in replication. During HDV replication, the coding sequence is translated into two delta antigens (HDAgs), a small and a large form (SDAg and LDAg), from the same reading frame; these are 195 and 214 amino acids in length, respectively [10,11]. Production of LDAg is through a process known as RNA editing, which is performed by cellular ADAR [12,13]; this converts the amber stop codon (UAG) of SDAg into a tryptophan codon (UGG), resulting in an extra 19 or 20 amino acids at the C-terminus of LDAg [14]. SDAg is essential for HDV replication while LDAg antagonizes the function of SDAg and is required to interact with HBsAg during virion assembly and maturation [15,16]. There is a CaaX-box (^211^CRPQ^214^, ^211^CTPQ^214^, and ^211^CTQQ^214 ^in various HDV genotypes, see Fig. 1A) at the C-terminus of LDAg, which acts as a signal of isoprenylation. Mutation of the isoprenylation signal of LDAg leads to a failure of virion assembly and secretion [17-19].

In addition to the isoprenylation signal sequence, a nuclear exporting signal (NES) has also been identified at the C-terminus of LDAg [20]. Within the common 195-amino-acid sequence of SDAg and LDAg, two putative leucine-zipper motifs, an RNA binding motif, and two nuclear localization signals have been identified [21-24]. Both SDAg and LDAg are phosphoprotiens with a different degree of modification [25]. After they have been either phosphorylated by PKC [26], CKII [18,26], PKR [27] or ERK1/2 [28], they affect HDV replication or are targeted to SC-35 speckles [18,26-28]. Both acetylation of HDAg at lysine-72 and methylation of SDAg at arginine-13 have also been demonstrated to influence HDV replication [29-31]. Conservation of these post-translational sites of HDAg among all known HDV genotypes suggests that the cellular enzymes responsible for the post-translational modifications of HDAg are at least partial if not all involved in HDV replication.

Co-infection and super-infection of HDV with HBV usually cause more severe liver disease than an HBV single infection [32]. The various HDV genotypes show different geographical distributions and are associated with different disease patterns [7,33]. HDV genotype I is distributed world-wide and has been linked to a wide spectrum of diseases, ranging from fulminant hepatitis to asymptomatic chronic liver disease. Genotype II is found mainly in Asia, including Japan, Taiwan and Siberia, and seems to give rise to a less severe disease than genotype I. Genotype III is mainly found in the north part of South America and produces a severe form of fulminant hepatitis [33]. The mechanism of HDV pathogenesis would seem to result from complicated interactions between HDV, HBV, and/or host factors and is not completely understood.

Two recent studies have indicated that LDAg rather than SDAg might play a significant role in HDV pathogenesis [34,35]. One study demonstrated a direct binding of LDAg to Smad-3, which modulates TGF-β signaling to activate plasminogen activator inhibitor-1 expression and c-Jun-induced signal cascades; this would seem to lead to liver cirrhosis [35]. The other study demonstrated that the cytoplasmic form of LDAg binds to the clathrin heavy chain (CHC) and further suggested that this LDAg-CHC interaction is required for HDV assembly. Furthermore, the LDAg-CHC interaction would seem to interfere with the clathrin-mediated endocytosis and exocytosis, which might finally lead to the hepatocytes damage [34]. However, the clathrin-box (^199^LFPAD^203^) identified in the LDAg of genotype I is not conserved in the same position in genotype II and III (Fig. 1A). This study was carried out with the aim of verifying whether the clathrin-binding activity is conserved across the three major genotypes of LDAgs.

## Results

The alignment of LDAg amino acid sequences from the three HDV genotypes indicates two features: 1) the common 195-amino-acid sequence shared by HDAg is conserved because it contains many functional motifs and post-translational modification sites, which are important for viral replication and maturation, and 2) the unique C-terminal sequence of LDAg is highly variable and differs in numbers of amino acids, in which genotype I and II have 19 residues while genotype III has 20 residues (Fig. 1A). Additionally, the presence of consensus sequence of the clathrin box (LϕxϕD/E) [36,37] and its location are also different at the C-terminus of LDAg (Fig. 1A). There is a clathrin box located at amino acids 199–203 in genotype I and a clathrin box at amino acids 206–210 in genotype II while no clathrin box in genotype III. Further alignment of all known HDV sequences by the C-terminal end of LDAg shows that when 43 isolates across clades 1 to 8 were compared, they could be divided into three different groups. Group 1 contains a clathrin box [LFP(A,S,V)D] located at amino acids 199–203 as found in genotype I. Group 2 contains a clathrin box (LPLLE) located at amino acids 206–210 as found in genotype II. Finally, group 3 contains no clathrin box as found in genotype III (Table 1). Among 43 HDV isolates, 13 sequences belong to group 1, 26 belong to group 2 and 4 are group 3.

**Table 1:** Three groups of the C-terminus of LDAg from 43 HDV isolates based on the presence and location of clathrin box

**Clades**	**Isolate**	**Extra a.a. residues**	**Clathrin Box**
**HDV-1**	Ethiopia	WDILFPSDPPFSPQS-CRPQ	LFP(A,S,V)D group 1
	US-1	WDILFPADPPSSPQS-CRPQ	
	US-2	WDILFPADPPFSPQS-CRPQ	
	Nagasaki-2	WDILFPVDPPFSPQS-CRPQ	
	Taiwan	WDLLFPADPPFSPQS-CRPQ	
	TW2667	WDILFPADPPFSPQS-CRPQ	
	China	WDILFPADPPFSPQS-CRPQ	
	Italy	WDILFPADPPFSPQS-CRPQ	
	Nauru	WDILFPSDPPFSPQS-CRPQ	
	Cagliari	WDLLFPADPPFSPQS-CRPQ	
	HDV-Iran	WDILFPSDPPFSPQS-CRPQ	
	Lebanon	WDILFPSDPPFSPQS-CRPQ	
	Somalia	WDILFPSDPPFSPQS-CRPQ	

**HDV-2**	Japan	WVSPSPPQQRLPLLE-CTPQ	LPLLE group 2
	Taiwan-3	WVHPSPPQQRLPLLE-CTPQ	
	Miyako-37	WVRPSPPQQRLPLLE-CTPQ	
	TW2476	WVRPSPPQQRLPLLE-CTPQ	
	Yakut-26	WVNPVPPGQRLPLLE-CTPQ	
	Yakut-62	WVNPAPPGQRLPLLE-CTPQ	

**HDV-3**	Peru-1	WYGFTPPPPGYYWVPGCTQQ	None group 3
	VnzD8624	WYGFTPPPPGYYWVPGCTQQ	
	VnzD8375	WYGFTPPPPGYYWVPGCTQQ	
	VnzD8349	WYGFTPPPPGYYWVPGCTQQ	

**HDV-4**	Miyako	WVDPGRPSPRLPLLE-CTPQ	LPLLE group 2
	L215	WVGPGSPSPRFPLLE-CTPQ	
	Miyako-36	WVNPQPPPPRLPLLE-CTPQ	
	Taiwan-Tw-2b	WVSPQPPPPRLPLLE-CTPQ	
	AF209859	WVSPQPPPPRLPLLE-CTPQ	
	Tokyo	WVNPQPPPPRLPLLE-CTPQ	

**HDV-5**	dFr2600	WVNPGPXPPRLPLLE-CTPQ	LPLLE group 2
	dFr2005	WVSPGSPSPRLPLLE-CTPQ	
	dFr47	WVNPGPRPPRLPLLE-CTPQ	
	dFr910	WVDPGPRPPRLPLLE-CTPQ	
	dFr73	WVSPGAPSPRLPLLE-CTPQ	
	dFr2703	WVSPXPXPPRLPLLE-CTPQ	

**HDV-6**	dFr48	WGNTPPRPPRLPLLE-CTPQ	LPLLE group 2
	dFr2139	WGNGPPRSPRLPLLE-CTPQ	
	dFr2627	WGNTPPRPPRLPLLE-CTPQ	

**HDV-7**	dFr45	WGNTPPRPPRLPLLE-CTPQ	LPLLE group 2
	dFr2158	WGPSPTPPPRLPLLE-CTPQ	

**HDV-8**	dFr2072	WGQSPPPPPRLPLLE-CTPQ	LPLLE group 2
	dFr644	WGQRPPPPPRLPLLE-CTPQ	
	dFr2736	WGQQPPPPPRLPLLE-CTPQ	

**Figure 2:**
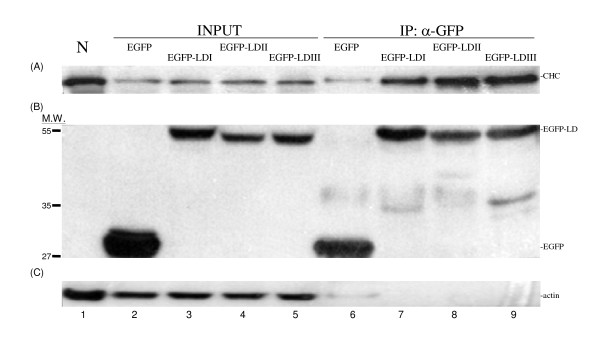
**An *in vivo *binding assay of EGFP-LDs to clathrin**. HuH-7 cells were transfected by various plasmids, as indicated above the blot, to allow expression of the EGFP-LDs. The total cell lysate and anti-GFP precipitated proteins were analyzed for CHC (2A), GFP (2B), and actin (2C) by Western blotting. NC (lane 1) represents non-transfection HuH-7 cells as a control. Lanes 2 to 5 are total cell lysates and lanes 6 to 9 are the anti-GFP immunoprecipitated proteins.

If clathrin-binding by LDAg is important to the HDV life-cycle, this property should be conserved across all genotypes of HDV. To answer this question, we designed *in vivo *CHC binding experiments to test whether exogenously expressed EGFP-LD can bind to endogenous CHC or not. HuH-7 cells, a human hepatoma cell line, were first transfected with the pEGFP-LD series of plasmids and treated with TNF-α at 24 h post-transfection for 2 h so that the EGFP-LD would behave like authentic LDAg [38] and be translocated from the nucleus to cytoplasm after the TNF-α treatment [39]. The transfected cells were immunoprecipitated by anti-GFP. This was followed by Western blot analysis, which showed that CHC was co-precipitated by all three fusion proteins, EGFP-LDI, EGFP-LDII, and EGFP-LDIII (Fig. 2, lanes 7–9), but not by EGFP (Fig. 2, lane 6). The amount of CHC co-precipitated with EGFP-LDI, -LDII, and LD-III was quantified after normalization and found to have a ratio of 1: 2.6: 2.2, respectively, averaged across three independent experiments. This indicates that LDAg binding to CHC is conserved and that the binding capacity varies across the three genotypes of HDV.

**Figure 3:**
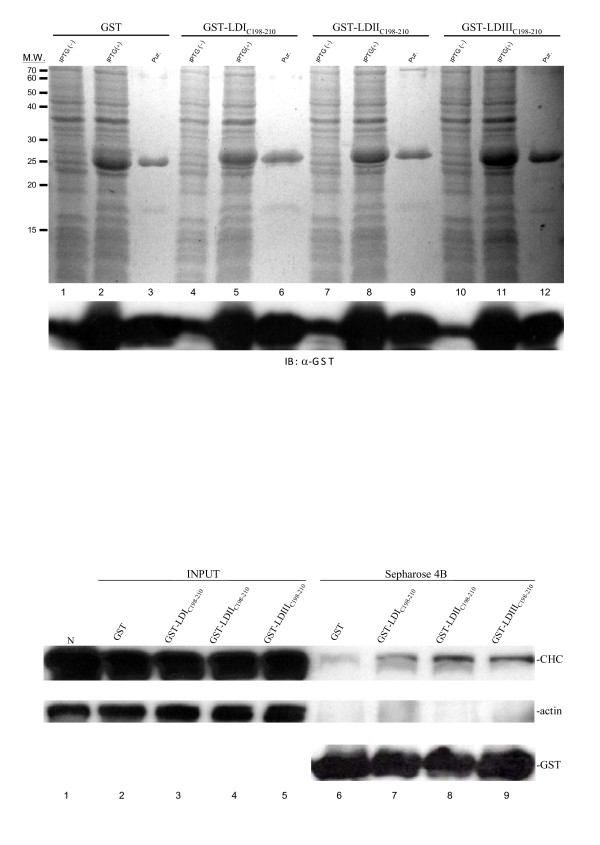
**An *in vitro *binding assay of LDAg peptides to HuH-7 cellular clathrin**. (A) Expression and purification of GST and the GST fusion proteins with the 13-amino-acid peptides of LDAg in *E. coli*. Total *E. coli *protein profiles are in even number lanes and purified proteins are in odd number lanes. The top gel was stained with Coomassie blue and the bottom gel was analyzed for GST. GST fusion proteins from different LDAg genotypes are indicated above the gel. Protein makers are shown to the left of the gel. (B) Western blot analysis for *E. coli *expressed LDAg peptide binding to CHC from a HuH-7 cell lysate. The top gel was detected for CHC, middle gel for actin, and bottom gel for GST. The GST and GST fusion proteins used for the binding assay are indicated above the gel. Lanes 1 to 5 are total cell lysate and lanes 6–9 are the GST-pull downs.

To verify the CHC binding by LDAg of genotype II and III is indeed through the C-terminus of LDAg as demonstrated by genotype I [34], we expressed the 13-amino-acid peptide (amino acid positions 198 to 210) of LDAg which is fused to GST, in *E. coli *(Fig. 3A). Purified GST-LD_C(198–210) _proteins bound to glutathione-Sepharose beads were then incubated with HuH-7 cell lysate for 16 h. The cell lysate and GST fusion protein mixtures were then spun down and analyzed by Western blotting. The results showed that the full-length CHC from HuH-7 cells was able to bind to the GST fusion proteins containing the peptide from the three LDAg (Fig. 3B, lanes 7–9) but not to GST only (Fig. 3B, lane 6). The normalized amount of CHC binding to the LDAg terminus of genotype I, II and III was in a ratio of 1: 1.6: 1.3, respectively, averaged across three independent experiments. Thus, the binding capacity of genotype II and III was lower than that found by the *in vivo *experiments as shown in Fig. 2. Nevertheless, this result indicates that the 13-amino-acid peptide of the GST-LDIII_C(198–210)_protein, which is without an identifiable clathrin-box, was still able to bind to full-length CHC and that this binding to CHC was stronger than the GST-LDI_C(198–210) _protein that does contain a clathrin box.

**Figure 4:**
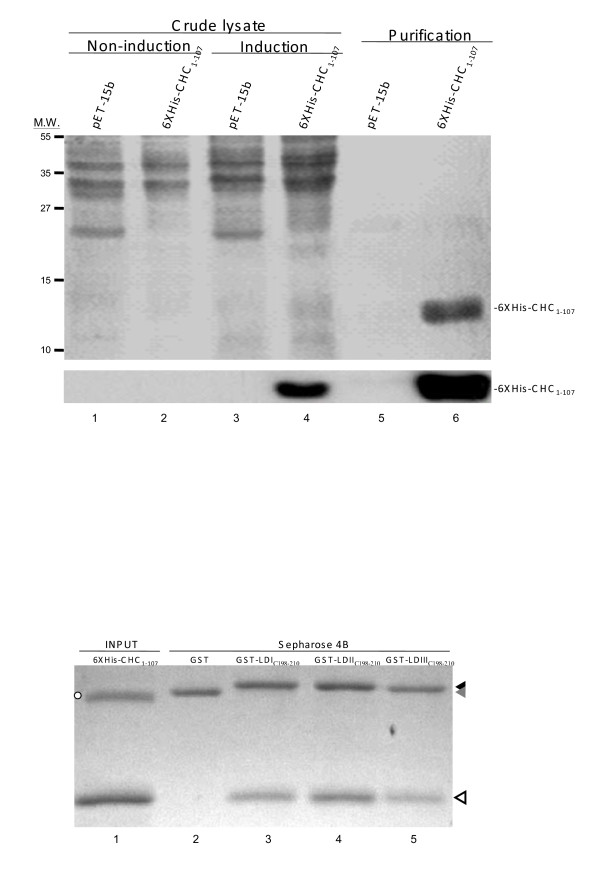
**An *in vitro *binding assay of LDAg peptides to CHC_(1–107) _fragment produced from *E. coli***. (A) Expression profile and purification of the fragment of CHC in *E. coli*. The top gel was stained with Coomassie blue and the bottom gel was detected by anti-His antibody. The protein makers are indicated to the left of the gel. (B) *E. coli *expressed GST and GST fusion proteins as shown in Fig. 3 were incubated with purified CHC as shown in (A). The GST pull-down products were analyzed by SDS-PAGE and silver stained. The proteins used for the pull-down are indicated above the gel and their migration positions are indicated by a black arrowhead (GST-LD), a gray arrowhead (GST) and a white arrowhead (CHC). Open circle (lane 1) indicates an unknown protein co-purified with CHC from *E. coli*.

Since no consensus sequence of a clathrin box is present in the C-terminus of genotype III LDAg, we further verified whether the CHC fragment (amino acid 1 to 107) is the specific binding site for the peptide (^198^FTPPPPGYYWVPG^210^) of genotype III LDAg. A fragment of CHC fused with hexahistidine was expressed in *E. coli *(Fig. 4A) and then purified by nickel beads. The glutathione-Sepharose beads bound with GST fusion proteins containing the 13-amino-acid peptide of LDAg from the various genotypes was then individually incubated with 6XHis-CHC_(1–107) _for 16 h. The spun down mixtures were separated by SDS-PADE and stained by silver. The results showed that the fragment of CHC_(1–107) _was pulled down by beads containing GST fusion protein (Fig. 4B, lanes 3–5) but not by those containing GST only (Fig. 4B, lane 2). The amount of CHC fragment pulled down by GST-LDI_C(198–210)_, GST-LDII_C(198–210)_, and GST-LDIII_C(198–210) _was averaged from three independent experiments and showed the ratio 1: 1.4: 0.7. Taken all these results together, it would seem that the LDAg of genotype II, either as a full-length protein or a 13-amino-acid peptide, had the highest binding capacity to the full-length or 107-amino-acid peptide of CHC as comparing three genotypes. The LDAg of genotype III had the second highest binding capacity to the full-length of CHC (Fig. 2A and Fig. 3B), however, had the lowest binding capacity to the fragment of CHC.

## Discussion

In this study, we have demonstrated that binding to CHC by the C-terminus of LDAg is conserved across all three major genotypes of HDV, although their amino acid sequences (^198^LLFPADPPFSPQS^210 ^in genotype I, ^198^GPSPPQQRLPLLE^210 ^in genotype II, and ^198^FTPPPPGYYWVPG^210 ^in genotype III) are highly variable in this region (Fig. 1A). Based on the variable sequences, the 43 HDV isolates analyzed can be classified into three groups (Table 1) and the three genotypes (I to III), described earlier in this study, each are represented by one of these groups.

It is reasonable to speculate that the CHC binding by the 13-amino-acid peptide of genotype II LDAg is through a putative clathrin box (LPLLE) that is located between amino acids 206 to 210. It has the highest binding capability to CHC among the three genotypes and this might be a result of a clathrin box at the most proximal end to the C-terminus. Whether such strong binding to CHC reduces genotype II LDAg's interaction with HBsAg and results in poorer packaging by HBsAg as found by Hsu et al. [40] is not determinable from these results. How the peptide of genotype III LDAg, lacking of a clathrin box, binds to CHC is also unclear. There are two possibilities. Firstly, the proline-rich stretch present in genotype III LDAg, which is similar to that of synaptojanin, might serve as a clathrin binding sequence [41]. Alternatively, cellular factors, such as AP-2, might form a complex with genotype III LDAg, through the ^205^YYWV^208 ^or ^206^YWVP^209 ^motif [41], and CHC. The results show a weaker binding affinity to the CHC_(1–107) _fragment produced from *E. coli *than to the full-length of CHC from the HuH-7 cell lysate by the peptide of genotype III LDAg (Fig. 4B vs. Fig. 3B). This suggests that firstly, cell factors are possibly involved in the genotype III LDAg binding to CHC and/or secondly, the fragment of CHC might have a different conformation from the full-length CHC. This change conformation might be less favorable to genotype III LDAg peptide binding. Based on the above results, we believe a further investigation to elucidate the hypotheses is warranted.

It has been demonstrated that the LDAg is required for interacting with HBsAgs to form mature virions or empty particles containing LDAg [16,42]. Questions on where and how HDV virions and empty particles are produced and mature remain unanswered. It is possible that the interaction between clathrin and the C-terminus of LDAg may assist HDV budding into the lumen of various intracellular membranes, such as ER or Golgi apparatus. Thus the LDAg may act as a late (L) domain similar to the situation during budding of many retroviruses [36,37]. There are three known classes of L domains for retroviruses, namely, PTAP, PPXY, and YXXL. The ^202^PPGY^205 ^sequence is present in genotype III LDAg, but is not present in genotype I and II, and this fits well into the second class of L domain. Whether this sequence functions as an L domain for genotype III HDV budding remains to be tested.

The fact that clathrin binding by LDAg is conserved across all genotypes must reflect its biological importance to the HDV life-cycle. We therefore postulate that any sequence at the C-terminus of LDAg that is able to bind to clathrin will be selected during evolution and this explains the great diversity across the various genotypes. Whether LDAg binding to clathrin, which may impair the normal functioning of clathrin, is one of causes of HDV pathogenesis as suggested by Huang et al. [34] remains to be elucidated, although many clathrin-associated diseases have been reported [43,44].

## Methods

### Plasmids used in this study

The cDNAs coding LDAg from different HDV genotypes, American strain of genotype I (accession number M28267), Taiwan-3 strain of genotype II (accession number U19598), and Peru-1 strain of genotype III (accession number L22063), were used in this study and have been described previously [33,45,46]. In this study, plasmids were divided into two groups: one for transfection into human hepatoma cells, HuH-7 [47], and the other for transformation into *E. coli *for protein expression. For transfection studies, the three genotypes of the LDAg coding sequence were constructed into pEGFP-C3 using *Bgl*II and *Eco*RI cloning sites as described previously [38]. The plasmids for protein expression were constructed by the insertion of synthetic oligonucleotides (Fig. 1B) downstream of GST in pGEX-4T1 using *Eco*RI and *Sal*I sites; this gave pGST-LDI_C(198–210)_, pGST-LDII_C(198–210)_, and pGST-LDIII_C(198–210)_, respectively. These clones were first selected checking for the presence of the *Eco*RV site and then verified by nucleotide sequencing. The cDNA fragment of human clathrin heavy chain containing amino acid 1 to 107 was amplified by RT-PCR and then inserted downstream of hexahistidine-tag sequence of pET-15b to give p6XHis-CHC_(1–107)_; which was used to express 6XHis-CHC_(1–107) _protein in *E. coli*. The plasmid was verified by nucleotide sequencing.

### Antibodies and affinity beads

The antibodies used in this study were purchased from different commercial companies. Anti-GFP was obtained from Clontech (California, USA) and Chemicon (California, USA). Anti-GST and anti-6XHis were bought from LTK BioLaboratories (Taoyuan, Taiwan). Anti-clathrin heavy chain was purchased from BD (California, USA). Anti-actin was obtained from Novus Biologicals (Colorado, USA). The secondary antibody conjugated with horseradish peroxidase was bought from Chemicon. Glutathione beads and nickel beads were purchased from GE (New Jersey, USA) and Qiagen (California, USA), respectively.

### Cell culture and plasmid transfection

HuH-7 is a fully-differentiated human hepatoma cell line [47] and was cultured at 37°C under 5% CO_2 _using Dulbecco's modified Eagle's medium supplement with 10% fetal bovine serum, penicillin (100 U/ml), streptomycin (100 μg/ml), and 1% non-essential amino acid. Cells at 60% confluence in a 10 cm Petri dish were transfected with 10 μg of plasmid by the calcium phosphate/DNA precipitation method or by adding lipofectamin 2000 (Invitrogen; California, USA). The transfection rate of each experiment was determined by the GFP expression under a fluorescence microscope.

### Protein expression in E. coli and purification

*E. coli *strain BL21 (DE3) was used to express GST and GST fusion proteins as well as 6XHis-CHC_(1–107)_. Bacteria were grown in LB medium supplement with 100 μg/ml ampicillin while vigorously shaken at 37°C until an O.D. 600 nm of 0.6 was reached. Production of protein was induced by the addition of 1 mM IPTG to the medium for 1 to 3 h. The resulting fusion proteins were further purified by one step binding to either glutathione affinity beads or nickel beads as appropriate and then analyzed by SDS-PAGE and Western blotting.

### Immunoprecipitation, GST-protein pull down and Westernblotting

At 24 h post-transfection with pEGFP-C3, pEGFP-LDI, pEGFP-LDII or pEGFP-LDIII into HuH-7 cells, the cells were treated with TNF-α (30 ng/ml) for 2 h [39]. Cells were lysed and immunoprecipitatd by anti-GFP. The precipitated proteins were fractionated by SDS-PAGE and electrotransferred onto PVDF membranes. The membrane was then incubated with anti-clathrin, anti-GFP, and anti-actin antibodies individually. After incubation with the secondary antibody conjugated with horseradish peroxidase, the blots were developed by enhanced chemiluminescence using a commercial kit (Pierce; Illinois, USA). The intensity of the protein bands was quantified by the program Image J software (NIH, Maryland, USA). In order to compare the pull-down efficiency of the different genotype LDAgs, the amount of CHC was normalization against actin and the amount pulled down by genotype I EGFP-LD was designated as 1. Three independent results were averaged and compared. Glutathione Sepharose 4B beads containing various GST fusion proteins were incubated with HuH-7 cell lysates or *E. coli *produced 6XHis-CHC_(1–107) _for 16 h at 4°C. After three washes with 0.05% Tween-20 in PBS (phosphate buffered saline), the proteins bound to the beads were analyzed and quantified by Western blot and Image J software as described above.

## Abbreviations

HBV: hepatitis B virus; HBsAg: surface antigen of HBV; HDV: hepatitis delta virus; HDAg: hepatitis delta antigen; LDAg: large delta antigen; SDAg: small delta antigen; CHC: clathrin heavy chain; EGFP: enhanced green fluorescence protein; GST: glutathione-*S*-transferase.

## Competing interests

The authors declare that they have no competing interests.

## Authors' contributions

YCW performed plasmid constructions and conducted experiments. CRH analyzed the 43 HDV sequences, performed experiments and prepared the figures. MC participated in discussion of results and revision of the manuscript. SJL participated in the design of the study and drafted the manuscript. All authors read and approved the manuscript.
